# Supplementary material for: Kinetochore-centrosome feedback linking CENP-E and Aurora kinases controls chromosome congression
Source: Nat Commun. 2025 Oct 21;16:9097. doi: 10.1038/s41467-025-64804-1 (PMC12540731; doi:10.1038/s41467-025-64804-1)
Supplement: Supplementary file 1 — Supplementary Information [file 41467_2025_64804_MOESM1_ESM.pdf]

Kinetochore-centrosome feedback linking CENP-E and Aurora kinases controls chromosome congression

Supplementary Information

Kruno Vukušić*, Iva M. Tolić*

Division of Molecular Biology, Ruđer Bošković Institute, Zagreb, Croatia

*Corresponding authors

E-mail: kvukusic@irb.hr, tolic@irb.hr

Supplementary Table 1. List of reagents, antibodies, cell lines, and software.

Reagent/Resource	Reference or Source	Identifier or Catalog Number
**Experimental Models**		
hTERT-RPE-1 cells stably expressing CENP-A-GFP	Alexey Khodjakov Lab, Wadsworth Center	N/A
hTERT-RPE-1 cells stably expressing both CENP-A-GFP and centrin1-GFP	Alexey Khodjakov Lab, Wadsworth Center	N/A
HeLa FlpIn cells expressing GFP-Hec1-9A	Geert Kops Lab, Hubrecht Institute	N/A
U2OS cells inducibly expressing GFP-CENP-E-T422	Marin Barišić Lab, Danish Cancer Institute	N/A
**Antibodies**		
Rabbit monoclonal Anti-NDC80	Sigma-Aldrich	HPA066330
Rat anti-alpha-tubulin YL1/2	Invitrogen	MA1-80017
Human anti-centromere (CREST) protein	Antibodies Incorporated	15-234
Rabbit polyclonal phospho-Aurora B (Thr232)	GeneTex	GTX85607
Rabbit polyclonal phospho-Hec1 (Ser55)	GeneTex	GTX70017
Rabbit monoclonal phospho-Aurora A (Thr288)/Aurora B (Thr232)/Aurora C (Thr198) (D12A11)	Cell Signaling Technology	2914T
Rabbit monoclonal phospho-Aurora A (Thr288) (C39D8)	Cell Signaling Technology	3079T
Rabbit monoclonal phopho-Dsn1 conjugated to Cy3 rabbit	Julie Welburn Lab, The University of Edinburgh	N/A
Rabbit monoclonal phopho-Knl1	Julie Welburn Lab, The University of Edinburgh	N/A

Donkey anti-rabbit IgG Alexa Fluor 647	Abcam	ab150075
Donkey anti-rabbit IgG Alexa Fluor 594	Abcam	ab150064
Donkey anti-rat IgG Alexa Fluor 647	Abcam	ab150155
Goat anti-human DyLight 594	Abcam	ab96909
Donkey anti-mouse IgG Alexa Fluor 594	Abcam	ab150108
Donkey anti-mouse IgG Alexa Fluor 647	Abcam	ab150107
Donkey anti-rat IgG Alexa Fluor 594	Abcam	ab150156
**Oligonucleotides and other sequence-based reagents**		
Human CENP-E ON-TARGETplus SMART pool siRNA	Dharmacon	L-003252-00-0010
Human custom-made 3’UTR HEC1 siRNA, oligo #3	Thermo Fisher Scientific	sequence 5′-CCCUGGGUCGUGUCAGGAA-3′
Human CENP-E 3’UTR siRNA	Marin Barišić Lab, Danish Cancer Institute	5′-CCACUAGAGUUGAAAGAUA-3′
control siRNA	Dharmacon	D-001810-10-05
**Chemicals, Enzymes and other reagents**		
MG-132	Merck	M7449
Lipofectamine RNAiMAX Reagent	Thermo	13778150
OPTI-MEM medium	Thermo	31985062
DMEM medium	Thermo	11995065
GSK-923295	MedChemExpress	HY-10299
Thymidine	Sigma	T1895
Monastrol	MedChemExpress	HY-101071A
Centrinone	MedChemExpress	HY-18682
MLN8054	MedChemExpress	HY-10180

ZM-447439	MedChemExpress	HY-10128
Alisertib (MLN8237)	MedChemExpress	HY-10971
TCS7010	MedChemExpress	HY-70061
Fetal bovine serum (FBS)	Thermo-Fisher	10270106
RO-3306	MedChemExpress	HY-12529
µ-Dish 35 mm, high Glass Bottom	Ibidi	81158
35 mm Dish 1.5 Coverslip 10 mm Glass Diameter Uncoated	Mattek	P35G-1.5-10-C
**Software**		
ImageJ (Fiji)	https://imagej.net/software/fiji/	N/A
Prairie View Imaging Software	Bruker	N/A
Imspector software	Abberior	N/A
ZEN 2.6 software	Zeiss	N/A
MatlabR2021a	MathWorks	
Matlab extension UnivarScatter	https://github.com/manulera/UnivarScatter	N/A
Matlab Statistics Toolbox R14	MathWorks	N/A
Adobe Illustrator CS6	Adobe Systems	N/A
Low Light Tracking Tool (v.0.10) ImageJ plugin	https://imagej.net/plugins/low-light-tracking-tool	N/A
**Microscopy systems**		
Expert Line easy3D STED microscope system	Aberior	N/A
LSM 800 confocal laser scanning microscope system	Zeiss	N/A
Lattice Lightsheet 7 microscope system	Zeiss	N/A

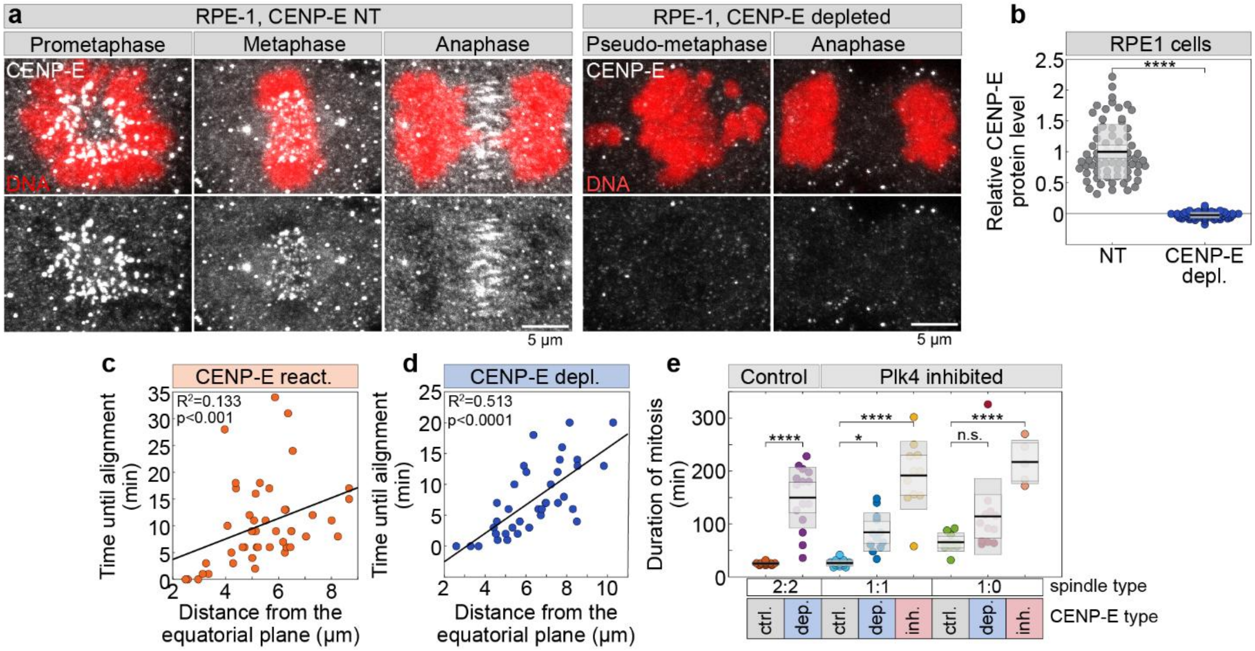

Supplementary Fig. 1. The speed of congression initiation depends on the distance of polar chromosomes from the centrosome. (**a**) Representative images of RPE-1 cells treated with non-targeting (NT) siRNAs (left) or CENP-E siRNA (right), immunostained with antibodies against CENP-E at different mitotic stages as indicated. Cells were wild-type (WT) and stained with DAPI (DNA, red). Top panels show merged channels; bottom panels show the CENP-E signal (gray). Images are maximum intensity projections. (**b**) Quantification of CENP-E protein levels after its depletion normalized to the NT control group. Colored points represent individual cells; black lines show the mean, with light and dark grey areas marking 95% confidence intervals for the mean and standard deviation, respectively. Numbers: 62 and 57 cells, each pooled from three independent biological replicates. (**c**, **d**) Correlation between the initial distance of polar kinetochore pairs to the equatorial plane and their total congression time, following washout of CENP-E inhibitor (c) or CENP-E depletion (d); lines represent regression fits. Numbers: (c) reactivated (18 cells, 86 kinetochore pairs), (d) CENP-E depleted (18 cells, 69 kinetochore pairs), each from more than 3 independent experiments. (**e**) Duration of mitosis, defined as time from nuclear envelope breakdown to anaphase onset, in cells with different numbers of centrioles and under different treatments, as indicated. Dispersion measures as in (b). Numbers of cells and independent biological replicates for (e) are given in Fig. 1. Statistics: (c, d) two-tailed t-test, (e) two-tailed ANOVA with post-hoc Tukey's HSD test. Symbols indicate: n.s., $P > 0.05$; *, $P \leq 0.05$; ****, $P \leq 0.0001$; reac., reactivated; depl., depleted; inh., inhibited; ctrl., control.

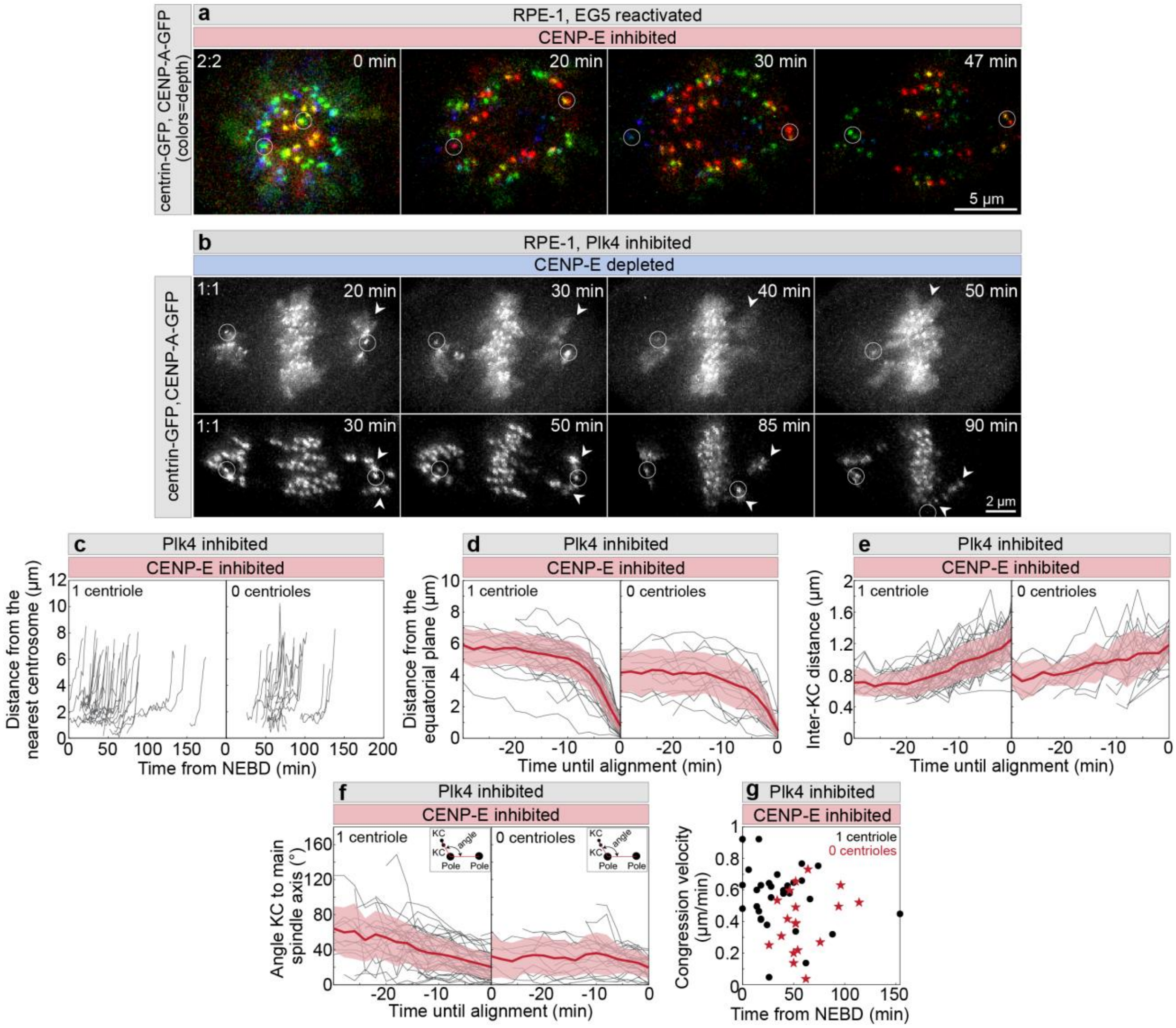

Supplementary Fig. 2. Chromosome movement dynamics during congression are similar in cells with different centriole numbers. (**a**) Representative images of an RPE-1 cell expressing CENP-A-GFP and centrin1-GFP (color-coded for depth), showing different time points after the washout of the Eg5 inhibitor monastrol and addition of 80 nM CENP-E inhibitor GSK923295. Time 0 marks the point of monastrol washout and GSK923295 addition. Centrioles are circled. (**b**) Representative time frames of two RPE-1 cells expressing CENP-A-GFP and centrin1-GFP (circled) (grey) following CENP-E depletion. Time 0 marks nuclear envelope breakdown (NEBD). Centrioles are circled in white. Arrowheads indicate polar chromosomes that align after centriole separation from the centrosome. (**c**–**f**) Quantification of kinetochore behavior from NEBD to successful alignment for initially polar kinetochore pairs in the presence of a CENP-E inhibitor: distance from the nearest spindle pole (c), distance to the equatorial plane (d), interkinetochore (inter-KC) distance (e), and angle relative to the main spindle axis (f; schematic shown in graph). Data compare chromosomes congressing from spindle poles with one centriole (left) versus those without centrioles (right). Thick lines in (d-f); means; shaded red areas, standard deviations. (**g**) Velocity of chromosome congression during the 6-minute period preceding full alignment, plotted

against the time from NEBD at which congression began, for polar chromosomes originating from poles with one centriole (black) or without centrioles (red). Numbers of cells and independent biological replicates for panels (c–g) are given in Fig. 1. Symbols indicate: KC, kinetochore; NEBD, nuclear envelope breakdown.

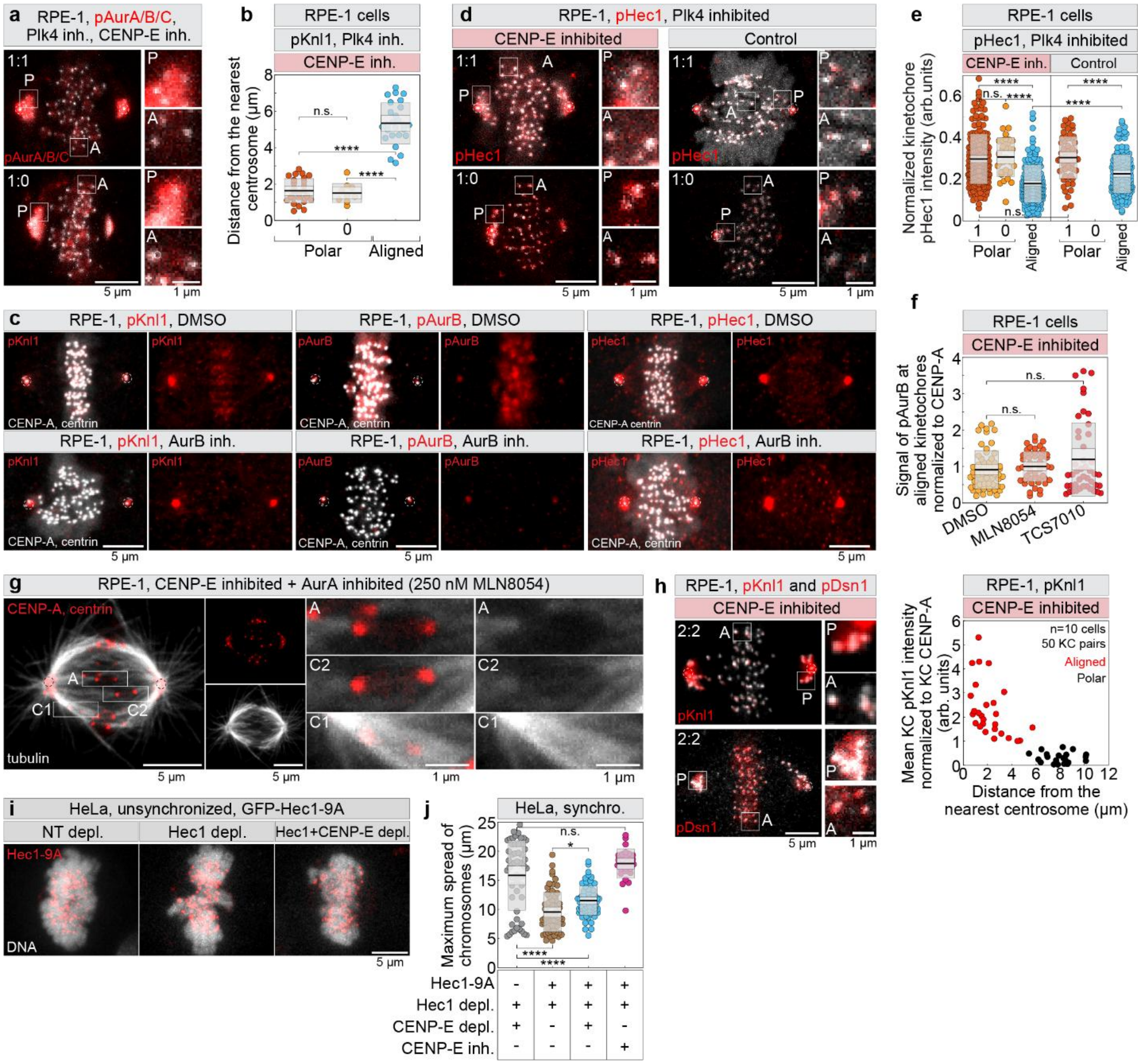

Supplementary Fig. 3. Aurora B-mediated phosphorylation of KMN network gradually declines during congression of polar chromosomes. (**a**) Representative images and enlarged insets of RPE-1 cells expressing CENP-A-GFP and Centrin1-GFP (circled) (grey), immunostained for pT288-Aurora A, pT232-Aurora B, and pT198-Aurora C (red), showing spindles with different centriole numbers after continuous 300 nM centrinone treatment and acute CENP-E inhibition as indicated. Images are maximum intensity projections. A, aligned; P, polar. (**b**) Distance from the nearest spindle pole for kinetochore groups identified in the pKnl1 immunolabelling experiment after CENP-E inhibition. Colored points represent individual cells; black lines show the mean, with light and dark grey areas marking 95% confidence intervals for the mean and standard deviation, respectively. Numbers: 62 kinetochore pairs pooled from two independent biological replicates. (**c**) Representative images of RPE-1 cells expressing CENP-A-GFP and Centrin1-GFP (grey, centrioles circled) and stained for phosphorylated kinetochore proteins (pS60-Knl1, pT232-Aurora B, pS55-Hec1) (red), with separated channels of immunostained proteins shown at right, following 15-minute treatment with DMSO or 3 µM Aurora B inhibitor ZM-447439. Images are

maximum projections. (**d**) Representative images of cells expressing CENP-A-GFP and centrin1-GFP (grey), with centrioles circled in white, immunostained for phosphorylated Hec1 (pS55-Hec1) (red) under the indicated conditions, with enlarged insets at right. Images are maximum projections. (**e**) Mean pS55-Hec1 intensity at kinetochores normalized to CENP-A intensity across treatments and centriole numbers. Dispersion measures as in (b). Numbers: 594 kinetochores, 67 cells, pooled from more than 3 independent biological replicates. (**f**) Levels of pT232-Aurora B on aligned kinetochores normalized to average CENP-A levels in CENP-E–inhibited cells after indicated acute treatments. Dispersion measures as in (b). Numbers of cells and independent biological replicates are given on Fig. 4. (**g**) Representative images of the RPE-1 cell expressing CENP-A-GFP and centrin1-GFP (red, centrioles circled), treated as indicated, showing congressing (C) and aligned (A) kinetochores with merged and separated channels, acquired by super-resolution Airyscan microscopy. Image is a deconvolved maximum projection of 5 z-planes, with α-tubulin stained in grey. (**h**) Representative images and enlarged insets of RPE-1 cells expressing CENP-A-GFP and centrin1-GFP (grey, centrioles circled), immunostained for pKnl1 (red) (top left) and pDsn1 (pS100, pS109-Dsn1, red) (bottom left) after CENP-E inhibition, and quantification of mean pKnl1 signal intensity versus spindle pole distance for polar and aligned kinetochore pairs (right). Experiment done in two independent biological replicates. Number of cells and kinetochores are given on the figure. (**i**) Representative examples of unsynchronized HeLa cells expressing GFP-Hec1-9A (red) with DNA stained by DAPI (grey) in treatments indicated on the top. (**j**) Maximum chromosome spread in synchronized HeLa cells under the indicated treatments, as shown in the legend. Dispersion measures as in (b). Numbers of cells and independent biological replicates for (j) are given on Fig. 5. Statistics: two-tailed ANOVA with post-hoc Tukey's HSD test. Symbols indicate: n.s., $P > 0.05$; *, $P \leq 0.05$; ****, $P \leq 0.0001$; inh., inhibited; depl., depleted; synchro., synchronized; KC, kinetochore.
