# Supplementary material for: Kinetochore-centrosome feedback linking CENP-E and Aurora kinases controls chromosome congression
Source: Nat Commun. 2025 Oct 21;16:9097. doi: 10.1038/s41467-025-64804-1 (PMC12540731; doi:10.1038/s41467-025-64804-1)
Supplement: Supplementary file 2 — Description of Additional Supplementary Files [file 41467_2025_64804_MOESM2_ESM.pdf]

Description of Additional Supplementary Files

Supplementary Movie 1. RPE-1 cells stably expressing CENP-A-GFP and Centrin1-GFP under continuous Plk4 inhibition, resulting in varying centriole numbers, following CENP-E inhibition or depletion. Cells were treated with a Plk4 inhibitor for 2 days to generate centrosomes with 1:1, 1:0, or 0:0 centriole configurations. CENP-E was either inhibited with a drug for 3 hours prior to imaging (left) or depleted via siRNA for 48 hours (right). Imaging started 3 hours after drug addition or 48 hours post-transfection. Kinetochores and centrioles are depth color-coded using a 16-color LUT from blue (bottom) to red (top).
