# Supplementary material for: Kinetochore-centrosome feedback linking CENP-E and Aurora kinases controls chromosome congression
Source: Nat Commun. 2025 Oct 21;16:9097. doi: 10.1038/s41467-025-64804-1 (PMC12540731; doi:10.1038/s41467-025-64804-1)
Supplement: Supplementary file 4 — Reporting Summary [file 41467_2025_64804_MOESM4_ESM.pdf]

nature portfolio

Corresponding author(s): Iva M. Tolić and Kruno Vukušić

Last updated by author(s): Sep 8, 2025

Reporting Summary

Nature Portfolio wishes to improve the reproducibility of the work that we publish. This form provides structure for consistency and transparency in reporting. For further information on Nature Portfolio policies, see our Editorial Policies and the Editorial Policy Checklist.

Statistics

For all statistical analyses, confirm that the following items are present in the figure legend, table legend, main text, or Methods section.

n/a	Confirmed	
☐	☒	The exact sample size (*n*) for each experimental group/condition, given as a discrete number and unit of measurement
☐	☒	A statement on whether measurements were taken from distinct samples or whether the same sample was measured repeatedly
☐	☒	The statistical test(s) used AND whether they are one- or two-sided<br>*Only common tests should be described solely by name; describe more complex techniques in the Methods section.*
☒	☐	A description of all covariates tested
☐	☒	A description of any assumptions or corrections, such as tests of normality and adjustment for multiple comparisons
☐	☒	A full description of the statistical parameters including central tendency (e.g. means) or other basic estimates (e.g. regression coefficient) AND variation (e.g. standard deviation) or associated estimates of uncertainty (e.g. confidence intervals)
☐	☒	For null hypothesis testing, the test statistic (e.g. *F*, *t*, *r*) with confidence intervals, effect sizes, degrees of freedom and *P* value noted<br>*Give P values as exact values whenever suitable.*
☒	☐	For Bayesian analysis, information on the choice of priors and Markov chain Monte Carlo settings
☒	☐	For hierarchical and complex designs, identification of the appropriate level for tests and full reporting of outcomes
☒	☐	Estimates of effect sizes (e.g. Cohen's *d*, Pearson's *r*), indicating how they were calculated

Our web collection on statistics for biologists contains articles on many of the points above.

Software and code

Policy information about availability of computer code

Data collection	Opterra confocal movies and images were acquired with Prairie View Imaging 5.4.64.500 software (Bruker).<br>STED movies and images were acquired with Imspector 16.3.16118-w2224-win64 software (Abberior Instruments).<br>Airyscan Zeiss LSM800 confocal movies were aquired with ZEN blue 3.5 software (Zeiss).<br>The Lattice Lightsheet 7 system movies were aquired with ZEN 3.7 software (Zeiss).
Data analysis	We used MATLAB R2021a and for data plotting and calculation of statistical parameters.<br>All movies and images were analyzed with Image J/Fiji.<br>Figures and schematic representations were assembled in Adobe Illustrator 2023 27.9 (Adobe Systems).

For manuscripts utilizing custom algorithms or software that are central to the research but not yet described in published literature, software must be made available to editors and reviewers. We strongly encourage code deposition in a community repository (e.g. GitHub). See the Nature Portfolio guidelines for submitting code & software for further information.

Data

Policy information about availability of data

All manuscripts must include a data availability statement. This statement should provide the following information, where applicable:

- Accession codes, unique identifiers, or web links for publicly available datasets
- A description of any restrictions on data availability
- For clinical datasets or third party data, please ensure that the statement adheres to our policy

All relevant data supporting the findings of this study are provided within the article and its Extended Data files. Raw images used in this work are available from the corresponding authors upon reasonable request.

Research involving human participants, their data, or biological material

Policy information about studies with human participants or human data. See also policy information about sex, gender (identity/presentation), and sexual orientation and race, ethnicity and racism.

Reporting on sex and gender	N/A
Reporting on race, ethnicity, or other socially relevant groupings	N/A
Population characteristics	N/A
Recruitment	N/A
Ethics oversight	N/A

Note that full information on the approval of the study protocol must also be provided in the manuscript.

Field-specific reporting

Please select the one below that is the best fit for your research. If you are not sure, read the appropriate sections before making your selection.

☒ Life sciences ☐ Behavioural & social sciences ☐ Ecological, evolutionary & environmental sciences

For a reference copy of the document with all sections, see nature.com/documents/nr-reporting-summary-flat.pdf

Life sciences study design

All studies must disclose on these points even when the disclosure is negative.

Sample size	Sample size was determined to provide a sufficient statistical power. Sample size and the number of biological replicates for each experiment are indicated in the figure captions.
Data exclusions	Only bipolar spindles were analyzed. Other exclusions were defined in the methods section.
Replication	Experiments were replicated in at least 3 independent experiments in majority of exeriments, unless otherwise noted in the figure captions and methods.
Randomization	Within each experimental regime, cell populations were assigned randomly to a siRNA, drug or control treatment. No other randomization was used.
Blinding	The investigators were not blinded to allocation during experiments and outcome evaluation.

Reporting for specific materials, systems and methods

We require information from authors about some types of materials, experimental systems and methods used in many studies. Here, indicate whether each material, system or method listed is relevant to your study. If you are not sure if a list item applies to your research, read the appropriate section before selecting a response.

Materials & experimental systems

n/a	Involved in the study
	☒ Antibodies
	☒ Eukaryotic cell lines
☒	☐ Palaeontology and archaeology
☒	☐ Animals and other organisms
☒	☐ Clinical data
☒	☐ Dual use research of concern
☒	☐ Plants

Methods

n/a	Involved in the study
☒	☐ ChIP-seq
☒	☐ Flow cytometry
☒	☐ MRI-based neuroimaging

Antibodies

Antibodies used

Primary antibodies used were:
- Rabbit monoclonal Anti-NDC80,
- Rat anti-alpha-tubulin YL1/2, Human anti-centromere (CREST) protein,
- Rabbit polyclonal phospho-Aurora B (Thr232),
- Rabbit polyclonal phospho-Hec1 (Ser55),
- Rabbit monoclonal phospho-Aurora A (Thr288)/Aurora B (Thr232)/Aurora C (Thr198) (D12A11),
- Rabbit monoclonal phospho-Aurora A (Thr288) (C39D8),
- Rabbit monoclonal phopho-Dsn1 conjugated to Cy3 rabbit,
- Rabbit monoclonal phopho-Knl1.

Secondary antibodies used were:
- Donkey anti-rabbit IgG Alexa Fluor 647
- Donkey anti-rabbit IgG Alexa Fluor 594
- Donkey anti-rat IgG Alexa Fluor 647
- Goat anti-human DyLight 594
- Donkey anti-mouse IgG Alexa Fluor 594
- Donkey anti-mouse IgG Alexa Fluor 647
- Donkey anti-rat IgG Alexa Fluor 594

Validation

All used antibodies are commercially available and validated before.

- Rabbit monoclonal Anti-NDC80 Sigma-Aldrich HPA066330
- Rat anti-alpha-tubulin YL1/2 Invitrogen MA1-80017
- Human anti-centromere (CREST) protein Antibodies Incorporated 15-234
- Rabbit polyclonal phospho-Aurora B (Thr232) GeneTex GTX85607
- Rabbit polyclonal phospho-Hec1 (Ser55) GeneTex GTX70017
- Rabbit monoclonal phospho-Aurora A (Thr288)/Aurora B (Thr232)/Aurora C (Thr198) (D12A11) Cell Signalling Technology 2914T
- Rabbit monoclonal phospho-Aurora A (Thr288) (C39D8) Cell Signaling Technology 3079T
- Rabbit monoclonal phopho-Dsn1 conjugated to Cy3 rabbit from Julie Welburn Lab, The University of Edinburgh
- Rabbit monoclonal phopho-Knl1 Julie Welburn Lab, The University of Edinburgh
- Donkey anti-rabbit IgG Alexa Fluor 647 Abcam ab150075
- Donkey anti-rabbit IgG Alexa Fluor 594 Abcam ab150064
- Donkey anti-rat IgG Alexa Fluor 647 Abcam ab150155
- Goat anti-human DyLight 594 Abcam ab96909
- Donkey anti-mouse IgG Alexa Fluor 594 Abcam ab150108
- Donkey anti-mouse IgG Alexa Fluor 647 Abcam ab150107
- Donkey anti-rat IgG Alexa Fluor 594 Abcam ab150156

Eukaryotic cell lines

Policy information about cell lines and Sex and Gender in Research

Cell line source(s)

hTERT-RPE1 cells stably expressing CENP-A-GFP, Alexey Khodjakov Lab, Wadsworth Center
hTERT-RPE1 cells stably expressing both CENP-A-GFP and centrin1-GFP Alexey Khodjakov Lab, Wadsworth Center
U2OS cells induciblely expressing GFP-CENP-E-T422 Marin Barišić lab, Danish Cancer Institute, Eibes et al., 2023.
HeLa FlpIn cells expressing GFP-Hec1-9A Geert Kops Lab, Hubrecht Institute, Etemad et al., 2015.

Authentication

None of the cell lines was authenticated by the authors.

Mycoplasma contamination

Cells were tested regularly for Mycoplasma contamination using DAPI and were found to be negative for Mycoplasma contamination.

Commonly misidentified lines
(See ICLAC register)

No commonly misidentified lines were used in this study.

Plants

Seed stocks	N/A
Novel plant genotypes	N/A
Authentication	N/A
